# Effect of SARs-CoV-2 pandemic on infection prevention behaviors and bacterial burden of high touch surfaces in a medical/surgical setting

**DOI:** 10.1038/s41598-022-09548-4

**Published:** 2022-04-01

**Authors:** Marisol Resendiz, Dawn M. Blanchard, Michael B. Lustik, Timothy S. Horseman, Gordon F. West

**Affiliations:** 1 Center for Nursing Science and Clinical Inquiry, Tripler Army Medical Center, Honolulu, HI USA; 2 Department of Clinical Investigations, Tripler Army Medical Center, Honolulu, HI USA; 3 Center for Nursing Science and Clinical Inquiry, Madigan Army Medical Center, 9040 Jackson Ave, Tacoma, WA 98431 USA

**Keywords:** Microbiology, Health care, Medical research

## Abstract

This study aimed to determine the longitudinal efficacy of ultraviolet germicidal disinfection (UV-C) in a non-terminal disinfection context. Moreover, factors influencing enhanced infection prevention behaviors during the SARS-CoV-2 pandemic were evaluated. Sixty nursing staff from three medical/surgical wards in a large military hospital were recruited for a survey and microbiological sampling of high-touch surfaces (stethoscope, personal electronic device, common access card, and hospital ID badge) and portable medical equipment (wheelchairs and mobile commodes). Surveys included hand hygiene estimates, frequency/method of cleaning items of interest, perception of UV-C, and factors influencing the use of enhanced disinfection tools. Surveys and microbiological samples were performed prior to and after the installation of a rapid, automated ultraviolet disinfection enclosure for staff use. Both time points preceded the SARS-CoV-2 pandemic in the United States. A final survey/sampling time point was carried out eight months after the declaration of the COVID-19 pandemic. Participants’ hand hygiene frequency did not increase throughout the study, with > 80% reporting a minimum of 4 hand hygiene events per patient hour. The cleaning frequency of high-touch surfaces (non-clinical) but not portable medical equipment increased after installation of a UV-C disinfection tool and was sustained eight months into the COVID-19 pandemic. While a modest decrease in bacterial burden was observed after UV-C intervention, a more significant reduction was observed across all surfaces during pandemic time sampling, though no detectable decrease in pathogenic contamination was observed at either time point. Motivators of UV-C use included fear of SARS-CoV-2 contamination and transmission, ease of device use, and access to rapid, automated disinfection tools while deterrents reported included technical concerns, lack of time, and preference for other disinfection methods. Automated, rapid-cycle UV-C disinfection can be efficacious for high-touch surfaces not currently governed by infection prevention and control guidelines. The introduction of enhanced disinfection tools like UV-C can enhance the overall cleaning frequency and is correlated with mild decreases in bacterial burden of high-touch surfaces, this is enhanced during periods of heightened infection threat. Evidence from this study offers insights into the factors which prompt healthcare workers to internalize/dismiss enhanced infection prevention procedures.

## Introduction

The World Health Organization (WHO) declared a global pandemic on March 11th, 2020 termed Coronavirus disease 2019 (COVID-19). COVID-19, caused by the SARS-CoV-2 virus, continues to be a major public health concern. Reduction of SARS-CoV-2 transmission by employment of social distancing, universal masking, and in-home sheltering have been the preeminent strategy for disease mitigation.

Transmission of SARS-CoV-2 includes contamination of eyes, mouth, and nose by hands exposed to virus-colonized fomites. Importantly, the virus has been shown to remain active on inanimate surfaces for up to 9 days at 30 °C, necessitating enhanced cleaning procedures, particularly on high touch surfaces^1^. Coronaviruses contain lipid envelopes which can be broken down by most disinfectants. Specifically, 78–95% alcohol, 0.5% hydrogen peroxide, or 0.21% sodium hypochlorite can inactivate the virus within 1 min^2^. While ultraviolet germicidal disinfection (UV-C) has demonstrated efficacy against SARS-CoV-2, efficacy is dependent on a variety of factors including surface type, virus titre, virus medium, and inoculum size^3^.

Previous surveys of healthcare workers in China found that risk of outbreak and working in a high-risk area increased self-reported infection prevention behaviors^4^.

Similarly, a study of infection prevention behavior compliance among healthcare workers in COVID-19 treatment centers in Ghana found that hand hygiene compliance and use of personal protective equipment (PPE) was generally high but lower among non-clinical staff or staff with secondary level qualifications^5^. The infection prevention behaviors of nursing staff in the United States during the COVID-19 pandemic have not been thoroughly investigated. In this study, we evaluated environmental hygiene behaviors including disinfection frequency and hand hygiene by comparing self-reported responses before, at the onset of, and eight months into the COVID-19 pandemic in the United States. As a secondary measure of environmental hygiene, we sampled the bacterial bioburden on four high-touch, non-medical surfaces that are frequently handled by nursing staff at the same time points, using detection of three common hospital pathogens as indicators of other communicable disease.

## Methods

### Participant recruitment and design

Nursing staff (RN, LPNs, and medics) were recruited from three medical/surgical wards in a large military hospital in the Pacific. The demographics of these staff were homogenous and included active duty military, government civilians, contractors, and military reservists.

Survey and microbiological sampling took place between September 2019 and November 2020. Data collection occurred at three time points on each of the units: a baseline survey/sampling period, post-intervention survey/sampling period trailing the rollout of a UV-C disinfection device and infection prevention educational campaign by one month (to optimize user experience and novelty-driven device use), and survey/sampling period coinciding with the eighth month of the SARS-CoV-2 outbreak in the United States. One month was allocated for each timepoint per unit such that all baseline sampling was completed by Nov 2019 and all post-UVC sampling by March 2020. Due to hospital restrictions and participant loss, all post-SARS-CoV-2 sampling was completed within Nov 2020 for all units. Twenty volunteers were recruited from each participating unit during the baseline period and these same participants provided post-UV and post-SARS-CoV2 data. Exclusion criteria included (1) non-nursing staff and (2) anticipated departure from the unit in the next month. Participants were recruited on a first-come, first-serve basis until all 20 spots were filled. The Tripler Army Medical Center Human Research Protections Office determined that this study did not constitute human subjects research and was thus exempt from IRB review (TAMC 19S16).

### Microbiological sampling

Personal stethoscopes, mobile phones, hospital ID badges, and Common Access Cards (CAC) were sampled from each volunteer at each of the three time points. The flat surfaces from each item (front and back) were swabbed with a flocked e-swab (BD CultureSwab, BD Biosciences, San Jose, CA) and were transported in 1mL of Liquid Amies medium for culturing with an hour of collection. Stethoscopes were swabbed at the membrane, bell, and both earpieces, however, not all participants carried a personal stethoscope.

E-swabs were first cultured onto 5% sheep’s blood soy trypticase agar plates (BA; BD Biosciences, San Jose CA), and the remaining liquid media was used to inoculate a chromogenic plate selective for vancomycin-resistant Enterococci (VRE) (VRE Select, BioRad, Hercules, CA). While it can be assumed that the proportion of sample was higher for the blood agar plate inoculation, this was in line with the goal of enumeration for that plate without significantly hindering the detection of VRE. All plates were incubated for approximately 24 h at 37 °C in 5% CO_2_. After incubation, BA plates were enumerated using an automated colony counter (Protos3, Synbiosis, Frederick, MD) and non-bacterial artifacts were manually removed for improved accuracy. Colony counts greater than 300 colony forming units (CFUs) were recorded as 300 to preserve accuracy^6^.

After enumeration, colonies of interest were subcultured for identification based on morphology and appearance. In particular, colonies consistent with *S. aureus* were subjected to standard biochemical methods (i.e., catalase and Staphaurex latex agglutination (ThermoFisher Scientific, Waltham, MD)) and VITEK 2 (bioMérieux, Durham, NC) for confirmation of methicillin-resistant Staphylococcus aureus (MRSA) and antibiotic susceptibilities. Colonies demonstrating a positive chromogenic response (per manufacturer’s guidelines) were subcultured for catalase and PYR testing (ThermoFisher Scientific, Waltham, MD). Final confirmation and susceptibilities were performed on the VITEK 2 and VITEK MS (bioMérieux, Durham, NC), respectively.

### Ultraviolet and educational intervention

After baseline sampling was completed on each unit, one CleanSlate UV (CleanSlate UV, Buffalo, NY) benchtop device was installed near the charge desk area. This touch-free, rapid-cycle device allows the automated disinfection of high-touch items such as smartphones, tablets, pagers, badges, and other non-medical devices at rates as high as 99.99% (for MRSA) in just 20 s based on laboratory tests^7^. Devices were introduced with heavy instruction, engagement, and a weekly educational campaign which included discussion, handouts, demonstrations, and other activities deployed during staff huddle. The educational campaign covered topics including, hand hygiene, chain of infection, mechanics of germicidal reagents, etc. The educational portion of the intervention was terminated during the heightening of hospital restrictions in March 2020 due to the SARS-CoV-2 outbreak. While all staff were trained and encouraged to use the CleanSlate UV, only study participants were surveyed/sampled.

### Survey of infection prevention behaviors

As part of a larger study, participants were administered a survey at each sampling point. The survey included a section soliciting information regarding infection prevention behaviors, specifically, participants were asked to provide estimates of the frequency of hand hygiene (to include soap and water and alcohol-based sanitizers) per hour of patient care (denoted commonly as ‘per patient hour’). Further, the cleaning frequency of the high-touch surfaces of interest and the preferred cleaning methods for these items were solicited uniformly throughout all three timepoints. Answers for cleaning frequency were arranged in multiple choice on a scale ranging from rarely/never, monthly, weekly, daily, or more than once daily. Preferred cleaning method was also arranged in multiple choice, using the common hospital cleaning reagents such as Cavicide (quaternary ammonium), dry wipes, alcohol-based wipe, UV-C, or others with a write in-option. This format was used to determine UV-C frequency throughout the study period, however, direct evaluation of the user’s experience with the CleanSlate UV was included in the post-intervention survey. Moreover, a multiple choice question was added to determine the motivators/deterrents of UV-C device use at the post-SARS-CoV-2 timepoint. Additional information was solicited regarding staff’s interaction with contact precaution patients, hand hygiene, attitudes, and beliefs at all timepoints.

### Data analysis

Data analysis included a comparison of CFU counts across the items of interest and overall. Differences in cleaning frequency and preferred cleaning method were also evaluated. To assess for changes at the individual level, paired data was utilized. However, due to participant attrition, our initial sample of 60 participants was reduced by 14 for a total of 46 participants by the second time point and again by 23 at the third timepoint for a total of 23 (though for some surfaces sample size was lower due to participant not having the item). A total of Wilcoxon signed rank tests were used to evaluate differences in CFUs between pre and postintervention. A significance level of *p*<0.05 was used for all analyses. All analyses were conducted using SAS statistical software version 9.4 (SAS Institute, Inc., Cary, NC).

### Ethics approval and consent to participate

The TAMC Human Research Protections Office determined that this study did not constitute human subjects research and was thus exempt from IRB review (TAMC 19S16).

## Results

Hand Hygiene in the medical/surgical wards profiled in this study generally showed high (self-reported) rates of hand hygiene frequency per patient hour, with 80% reporting at least 4 episodes of hand hygiene per hour (Table 1). The introduction of an ultraviolet disinfection tool for the treatment of high touch surfaces (non-medical) was rolled out with an educational campaign that included frequent reminders of the importance of hygienic retrieval (achieved by hand sanitation). Despite this campaign, hand hygiene frequencies >4/h only increased by 10% in the post-intervention period. Eight months after the post-UV-C survey, 73% of participants reported >4/h frequencies.

**Table 1:** Hand hygiene frequency among nursing staff in a medical surgical ward after ultraviolet intervention and during viral outbreak.

How many times do you perform hand hygiene (including soap and water, antiseptic hand rubs, etc.) per hour during the workday (estimate average)?	Baseline		Post-UV		Post-SARS-CoV-2	
	*n*	%	*N*	%	*n*	%
0–3 times/hour	4	20	2	10	6	26
4–6 times/hour	5	25	6	30	7	30
7–10 times/hour	5	25	6	30	6	26
> 10 times/hour	6	30	6	30	4	17

**Figure 1:**
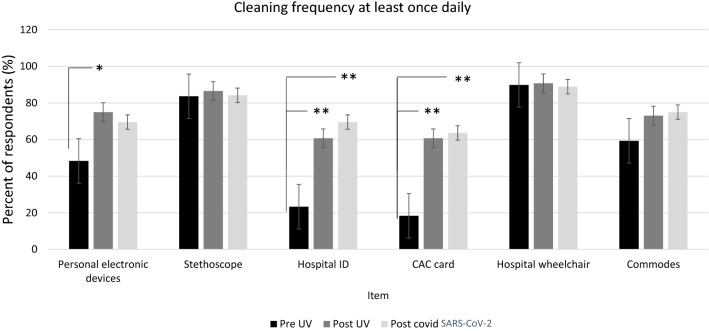
Percentage of nursing staff who performed disinfection of high-touch surfaces at least once daily. Baseline sampling: *n*=11 for CAC, *n*=14 for IDs, *n*=29 for PEDs, and *n*= 46 for stethoscopes. Post-UV sampling: *n*=34 for CAC, *n*=34 for IDs, *n*=34 for hands, *n*=42 for PEDs, and *n*=45 for stethoscopes. All post-SARS-CoV-2 sampling *n*=16 except for CAC (*n*=14) and commodes (*n*=12). CleanSlate UV was limited to include only cards, phones, and stethoscopes. Error bars denote Standard Error, **p≤0.001; *p≤0.05.

The proportion of study participants who cleaned high touch surfaces of interest at least once daily increased significantly after UV-C intervention for personal electronic devices, hospital IDs, and CAC cards. Further, hospital IDs and CAC cards also demonstrated a significant increase in the proportion of at least daily cleaning after the SARS-CoV-2 time point sampling (versus baseline). Neither stethoscopes, wheelchairs, or mobile commodes saw an increase in the proportion of daily cleaning. Interestingly, stethoscopes and wheelchairs had the highest percentage of daily cleaning at baseline (>80%). No item demonstrated a significant change in the proportion of daily cleaning from the post-UV timepoint to the post-SARS-CoV-2 timepoint (Figure 1).

**Figure 2:**
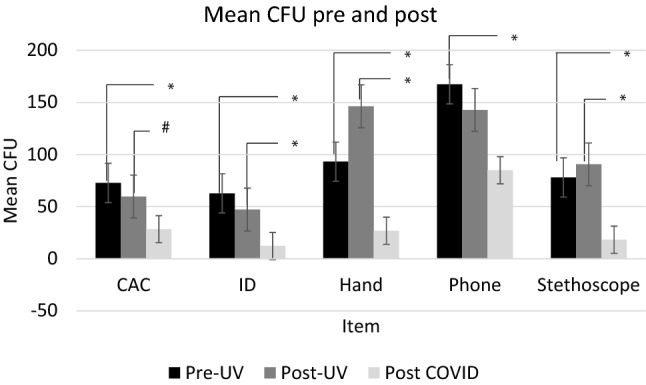
Bioburden of high-touch surfaces before and during a global outbreak of a novel coronavirus (SARS-CoV-2). Baseline sampling: *n*=59 for CAC, *n*=59 for IDs, *n*=31 for hands, *n*=60 for phones, and *n*= 43 for stethoscopes. Post-intervention sampling: *n*=57 for CAC, *n*=57 for IDs, *n*=34 for hands, *n*=56 for phones, and *n*=30 for stethoscopes. All post-outbreak sampling *n*=23 except for stethoscopes (*n*=20). Error bars denote Standard Error, *p≤0.001; #*p*≤0.05.

Three sampling events were performed to evaluate changes in bioburden before, during, and after circulation of SARS-CoV-2. Interestingly, three of the four surfaces as well as dominant hand samples exhibited significant decreases in bacterial colonization compared to the original post-intervention timepoint (aligned with the nascent stages of COVID-19, national case numbers on the last day of post-intervention sampling was 7327 cases and 115 deaths). All surfaces observed significant decreases in post-pandemic bacterial colonization compared to preintervention (baseline) sampling by comparison (Fig. 2).

Detection of MRSA/VRE positive samples were overall unaffected by the intervention. A total of 4 pathogenic colonies were identified across all units at baseline. After UV-C intervention, 7 pathogenic colonies were observed. During the post-SARS-CoV-2 timepoint, only 2 pathogenic colonies were identified. The majority of positive samples represent the common nosocomial pathogen, MRSA, with only two VRE isolated throughout the study. The vast majority of identified bacteria were predominantly commensal Gram-positive organisms.

Percentage reflects the proportion of respondents who selected either “a lot” or “a great” deal to perception of UV impact on work item disinfection, work environment cleanliness, and patient safety. All possible choices also included “a little” and “moderate”.

**Figure 3:**
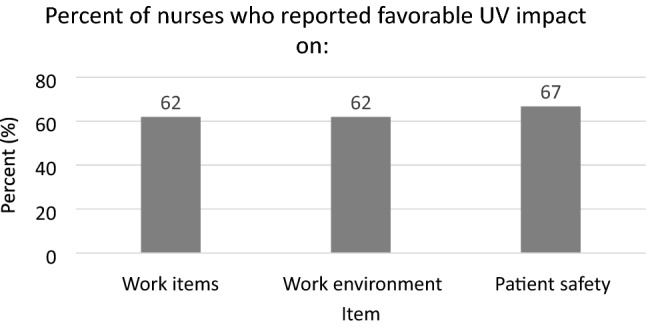
Staff perception of ultraviolet disinfection impact of work-related safety.

When staff were surveyed about their perception on the impact of UV-C intervention on the cleanliness of high touch surfaces handled frequently throughout the workday (CAC, hospital ID, PEDs, stethoscopes), 62% responded favorably. The same percentage of respondents perceived the addition of UV disinfection as having a favorable impact on the general work environment and 67% responded that UV-C had a favorable impact on patient safety (Figure 3).

The proportion of nursing staff treating patients on contact precautions during the survey period did not deviate from the general percentage (~ 40%) observed prior to SARS-CoV-2. However, the patient load of staff did increase for 36% of respondents while staying the same for the majority (55%). The greatest drivers of UV-C disinfection of high touch surfaces during the post-pandemic sampling period (8 months after nationwide lockdown measures were instated) revealed that risk of infection transmission and ease of use of the device were the two greatest factors (91% of respondents for each). Availability of the technology and increased vigilance of environmental hygiene were the next most prevalent influences, according to 77 and 64% of respondents, respectively. The greatest deterrent to the use of the UV-C device was technical issues related to device operation (36%). This was largely driven by a hardware issue that caused frequent arching electricity and bulb burnout on Unit 3 specifically. Despite this issue, which was eventually corrected, bulb replacement was continuously performed to minimize machine downtime. Aside from that technical issue, the largest deterrent of machine use was lack of time (32%) and preference for other cleaning agents (27%). These changes are outlined in Table 2.

**Table 2:** Motivators and deterrents of ultraviolet disinfection use eight months after the outbreak of SARS-CoV-2.

	*n*	%
**Today have you cared for contact patient?**		
No	14	61
Yes	9	39
**In last 90 days patient load has**		
Decreased	2	9
Increased	8	36
Stayed the same	12	55
**Which of the following have influenced your use of the UV device in the last 90 days Risk of transmission of infection**		
Ease of use	20	91
Availability of machine	20	91
Increased vigilance of env	17	77
Hygiene	14	64
Media/social discussion of UV	5	23
Leadership guidance/directive	4	18
Other	1	5
**Which of the following have deterred your use of the UV device in the last 90 days**		
Lack of time	7	32
Preference for other cleaning agents	6	27
Technical issues w/ device	9	41
Disbelief of germicidal effectiveness	1	5
Fear of exposure to UV light	1	5
Lack of guidance/directive	1	5
Ease of using alcohol in office	1	5
Forget machine is there	1	5

## Discussion

This study characterized the impact of an UV-C intervention longitudinally, with particular emphasis on utilization before and after an infection control crisis (COVID-19 pandemic). Additionally, we sought to better understand the drivers and deterrents of infection prevention behaviors of nursing staff. We describe that in a population with generally high performance of hand hygiene, further infection prevention initiatives such as ultraviolet tools and educational campaigns have minimal impact. While hand hygiene reports in healthcare settings have generally increased after the onset of SARS-CoV-2^8^, that was not the case in our population. This may be due to the different methods of hand hygiene quantification (the Roshan study observed hand hygiene compliance as defined by observation of hand hygiene performed divided by hand hygiene opportunity presented (i.e. before and after entering a patient’s room) between our studies. The Roshan study also reported hospital-wide rates of compliance compared to our paired-sampling approach which took place strictly in a mild-acuity medical/surgical section. Though a smaller sample was achieved, this method allowed us to more precisely characterize changes in staff behavior with control of interpersonal variability.

This longitudinal study revealed that environmental cleaning practices were improved after the addition of an ultraviolet disinfection tool but only for items lacking existing disinfection guidelines. Despite this, no further increases in cleaning practices (for the items of interest in this study) were detected eight months after the initial outbreak of SARS-CoV-2, though the initial increase was maintained. One study of cleaning practices among hospital staff during the COVID-19 pandemic reported high rates of environmental cleaning (~ 90%)^9^. It should be noted that environmental cleaning in that study was monitored by an infection prevention and control team (introducing observation bias) and focused entirely on terminal disinfection tasks which are heavily protocolized in contrast to the more voluntary cleaning/disinfection practices in our study. Another consideration for our results may be the phasing out of the educational component of the intervention after the onset of SARS-CoV-2 and the subsequent national lockdown in the United States.

Despite the lack of quantifiable increase in environmental cleaning behaviors (pertaining to the high touch surfaces of interest in our study) or hand hygiene after SARS-CoV-2, we did profile a significant reduction in bacterial contamination of multiple high touch surfaces (compared to the post-UV sampling period). This included the bacterial burden on nursing staff’s hands, stethoscopes, IDs, and access cards (though not PEDs). This suggests that even after accounting for the microbiological impact of ultraviolet disinfection, the COVID-19 pandemic brought about behavioral changes which contributed to a cleaner environment. These may include factors including but not limited to decreased transport of inpatients, decreased presence of visitors and non-essential staff, enhanced disinfection of clinical surfaces due to SARS-CoV-2 guidelines, and limited cross-contact between staff due to social distancing and mask wearing.

Despite quantifiable decreases in the bacterial bioburden of the high touch surfaces studied herein, the presence of common nosocomial pathogens (specifically MRSA, VRE, and *Clostridium difficile*) was relatively low and remained low throughout the study, regardless of intervention or SARS-CoV-2. This is not entirely uncommon as even throughout the COVID-19 pandemic, bacterial colonization was not a predictor of SARS-CoV-2 contamination in a study of high touch surfaces adjacent to COVID-19 hospitalization units^10^. It is unclear at this point whether the lack of intervention/coronavirus impact on common hospital pathogens in our study was truly due to low prevalence in our population or a methodological limitation such as too small of a sample.

Nursing staff welcomed the addition of an ultraviolet disinfection tool for personal use, and even after the novelty wore off, nearly two-thirds of staff favorably perceived the impact of the device on the cleanliness of high touch surfaces, the general work environment, and even patient safety. This is in agreement with previous surveys of staff attitudes and beliefs toward ultraviolet disinfection in clinical settings^11,12^. To date, however, little has been published regarding the motivators and deterrents of end-users toward ultraviolet disinfection tools. We investigated these along with the scope of our initial interest in ultraviolet disinfection technology however, in the wake of the SARS-CoV-2 outbreak, UV disinfection may serve as a proxy for several enhanced disinfection protocols/tools and the psycho-socialbiological factors that guide internalization of infection prevention practices. Here we learned that risk of infection transmission as well as ease of use of the tool overwhelmingly contributed as a motivating influence for use. Other prominent influences included the availability of disinfection technology and enhanced vigilance of environmental hygiene during the COVID-19 pandemic. In contrast, the biggest deterrents of UV disinfection included technical issues with the device (as mentioned previously this was largely driven by early internal issues with one particular UV enclosure on Unit 3). Technical issues notwithstanding, the other major factors hindering wider device use were lack of time and established preference for traditional cleaning methods (i.e. alcohol wipes, quaternary ammonium wipes). To our knowledge, this is the first survey of motivators/deterrents of voluntary, rapid UV disinfection technologies among nursing staff. This study thus provides insights for consideration prior to the implementation of staff directed supplementary disinfection tools and protocols. As expected, fear of infection transmission and heightened vigilance of environmental hygiene during the onset of SARS-CoV2 played influential roles in the internalization of UV as an enhanced infection prevention method. This echoes the findings of recent surveys of healthcare workers which documented as high as 31% of participants reporting anxiety, 11% fear, and 6% pessimism about the risk of contracting SARS-CoV-2^13^.

## Conclusion

The implementation of a voluntary, rapid ultraviolet disinfection enclosure was used in this study as a marker for enhanced infection prevention tools/protocols during the COVID-19 pandemic which persisted throughout the study period. While UV intervention prior to the outbreak significantly increased overall cleaning frequency on a number of high touch surfaces, items with existing disinfection guidelines (stethoscopes, wheelchairs, commodes) were unaffected. Interestingly the enhanced frequency of cleaning persisted on two of the three affected high touch surfaces (CAC cards and hospital IDs) as far as eight months into the COVID-19 pandemic. This corresponded with significant and persistent reductions in overall bacterial contamination of the surfaces, though rates of common, hospital multi-drug resistant organisms were not affected by either the UV intervention or the outbreak. While nearly two thirds of staff report highly favorable attitudes toward the impact of ultraviolet disinfection on environmental hygiene and patient safety, major use limitations were noted, specifically lack of time and preference for other cleaning agents. Another consideration for electronic based tools for enhanced infection prevention include the ease of use and possibility of technical malfunctions which may negatively influence users’ perception and utilization, as occurred in one unit in this study. Finally, we confirm in a US population of healthcare workers that fear of SARS-CoV-2 infection and transmission were prominent motivators for increased vigilance of environmental hygiene and increased use of UV disinfection.

## Data Availability

The datasets used and/or analyzed during the current study are available from the corresponding author on reasonable request.

## Abbreviations

CAC: Common access card
CDC: Centers for disease control
CFU: Colony forming units
COVID-19: Coronavirus disease 2019
LPN: Licensed practical nurse
MRSA: Methicillin resistant *Staphylococcus aureus*
PED: Personal electronic device
RN: Registered nurse
SARS-CoV-2: Severe acute respiratory syndrome coronavirus 2
UV (UV-C): Ultraviolet-C
VRE: Vancomycin resistant enterococci
WHO: World health organization
